# Isolated bladder metastasis causing large bowel obstruction: a case report of an atypical presentation of intussusception

**DOI:** 10.4076/1757-1626-2-7124

**Published:** 2009-06-11

**Authors:** Justin D Blasberg, Gary Schwartz, Jason A Mull, Eric Moore

**Affiliations:** 1 Department of Surgery, St Luke's-Roosevelt Hospital Center, University Hospital of Columbia University College of Physicians and SurgeonsNew York, NYUSA; 2 Department of Pathology, St Luke's-Roosevelt Hospital Center, University Hospital of Columbia University College of Physicians and SurgeonsNew York, NYUSA

## Abstract

Intussusception of the large bowel is a rare clinical entity. In adults, this pathology is usually associated with a malignant lead point and often requires operative management. Reported is the case of an 83-year-old female who was recently diagnosed with superficial bladder cancer (T1) treated by partial cystectomy. She presented 3 months post-operatively with an isolated mucosal metastasis of the transverse colon causing intussusception and large bowel obstruction. The patient was successfully treated by colonic resection with primary anastomosis. Histology was significant for a pedunculated sarcomatoid bladder carcinoma originating from the colonic mucosa with incomplete invasion of the bowel wall. An isolated mucosal metastasis of this variety has not been reported in the literature to date.

## Introduction

Metastatic disease from a primary genitourinary neoplasm has a typical pattern, with involvement of lymph nodes in the pelvis, liver, lung, bone, and adrenal glands. Histologic evaluation most commonly demonstrates transitional cell carcinoma, and less often squamous cell carcinoma. A sarcomatoid variant of bladder cancer is an extremely rare clinical entity with a reported incidence of only 0.31% [1]. These malignant tumors arise from atypical spindle cells with epithelial differentiation that may be demonstrated by immunohistochemical or ultrastructural studies [2]. Presented is the case of an elderly female who recently underwent partial cystectomy for sarcomatoid carcinoma of the bladder, and presented to the emergency room with clinical evidence of a bowel obstruction. Imaging revealed large bowel obstruction secondary to transverse colonic intussusception with a malignant lead point. Surgical resection ensued and histologic evaluation demonstrated a mucosal-based metastatic lesion consistent with the histology from her cystectomy specimen.

## Case presentation

An 83-year-old white female presented to the Emergency Department with a four-week history of progressively worsening epigastric abdominal pain and a four-day history of obstipation. Her past medical history was significant for hypertension and bladder cancer diagnosed 3 months prior to presentation, treated by partial cystectomy. Her pathology at that time was significant for a superficial bladder tumor with sarcomatoid features (T1), and was closely followed medical oncology. She had not received adjuvant therapy. The patient presented with persistent nausea without vomiting, fevers or chills, chest pain or shortness of breath. Her oral intake was decreased over the prior two weeks, worsening to complete food intolerance. She had no urinary complaints at presentation and denied any night sweats or weight loss. She had a 70-pack-year smoking history and drank alcohol on occasion.

On physical exam the patient appeared cachectic and uncomfortable but in no acute distress. She was afebrile, with a normal blood pressure but was slightly tachycardiac with a heart rate of 110 beats per minute. She was tachypnic with a respiratory rate of 23 breaths per minute with an oxygen saturation of 96% on room air. Her heart and lung exam was unremarkable, but her abdomen was grossly distended with hyperactive bowel sounds. She was tender primarily in the epigastrum with fullness in that region. There was no rebound tenderness, and she was voluntarily guarding in all four quadrants. No organomegaly was appreciated. Her lower midline cystectomy scar was noted. Rectal exam was not significant for any palpable masses and fecal occult blood testing was negative. Laboratory values were within normal limits and her chest X-ray was unremarkable.

**Figure 1.**
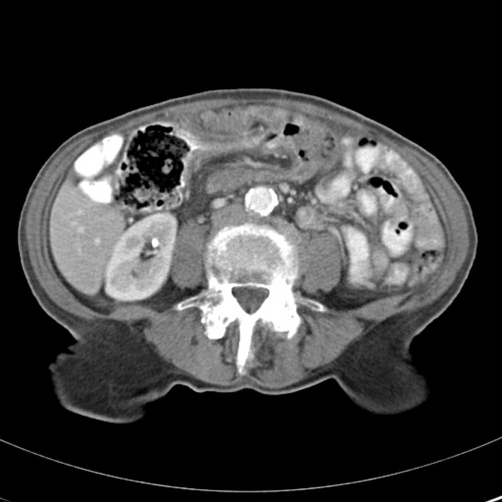
CT Scan abdomen with evidence of intussusception at the transverse colon.

A computed tomography scan of her abdomen with intravenous contrast demonstrated evidence of intussusception at the transverse colon with a 2 × 2 cm lead point characterized as a soft tissue density. There was appreciable thickening of the descending colon as well as para-aortic lymph nodes measuring 7-10 mm (Figure 1). The patient was admitted to the surgical service and prepared for an exploratory laparotomy.

### Operative Course

In the operating room a rigid proctoscopy was performed demonstrating normal mucosa up to 20 cm. The patient was prepped in standard surgical fashion and a midline incision was made. Evaluation of the proximal transverse colon revealed an intussusception involving a 6-8 cm segment. The remaining colon appeared normal without appreciable masses or thickening. A sleeve colectomy containing the segment was initially performed and opened in the operating room. No serosal involvement of the specimen was noted. Within the colon, a 2 × 5 cm pedunculated hard mass appeared to arise from the mucosa. Frozen section assessment was unable to determine a pathologic origin for the submitted tissue, including whether cells had a benign or malignant appearance. Due to the high suspicion for an underlying malignant process, a completion right hemicolectomy was performed. There was no evidence of metastatic disease on any serosal surfaces, nor within the liver. The remainder of the operation was uneventful, and the patient extubated and taken to the recovery room. She was monitored in the intensive care unit for 24 hours. Her diet was advance postoperative day three and she was discharged to subacute rehabilitation on post-operative day seven.

### Pathology

**Figure 2.**
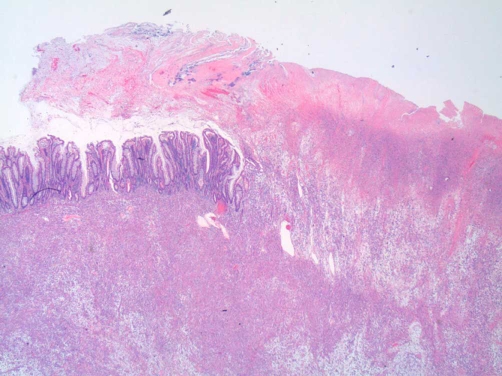
Low power colectomy specimen displaying tumor replacement of the normal colonic mucosa with protrudes into the lumen.

**Figure 3.**
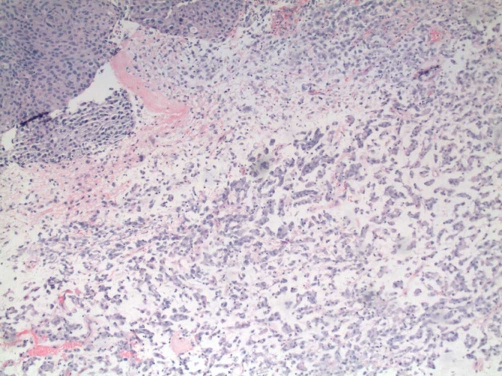
Area of original biopsy showing traditional urothelial carcinoma (upper left) and poorly-differentiated/sarcomatoid area (lower right).

**Figure 4.**
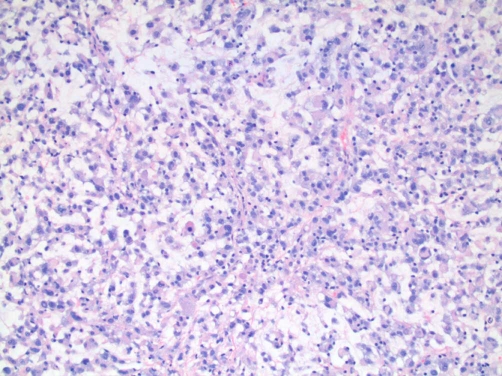
Metastasis specimen showing poorly-differentiated/sarcomatoid area.

**Figure 5.**
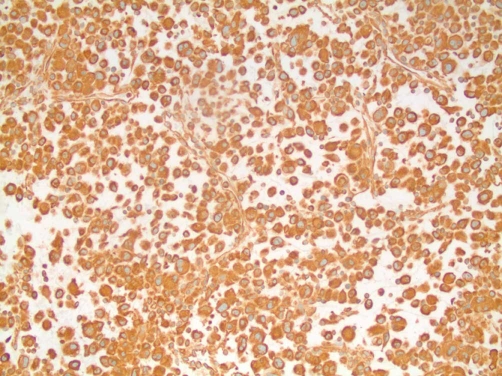
Strong, diffuse staining for vimentin supporting sarcomatoid differentiation.

Pathologic assessment of the resection specimen was remarkable for a mucosal-based lesion without gross invasion of the colonic wall. Twenty-five lymph nodes were included with the specimen, all negative for metastatic disease. Histological evaluation revealed a mucosal lesion, sarcomatoid in appearance, with incomplete invasion of the colonic wall and no serosal involvement (Figures 2-4). Comparison was made to the prior cystectomy specimen, with complete histological correlation. Immunohistochemistry and staining was significant for the presence of vimentin (Figure 5), highly specific for a sarcomatoid malignancy.

## Discussion

Intussusception in adult patients is relatively rare, representing only 5% of all cases of intussusception and less than 5% of all cases of bowel obstruction [3]. Incidence in the adult versus the pediatric population differs in both etiology and management. Whereas pediatric cases are predominantly benign and managed non-operatively with hydrostatic reduction, adult intussusception is the result of a malignant lead point in approximately 65% of cases [4] mandating surgical management.

Anatomic locations of intussusception include entero-enteric, colo-colic, and ileo-colic, and the differential diagnosis for the underlying etiology can be made upon location alone. Small bowel intussusception can be due to adhesions, Meckel's diverticulum, inflammatory bowel disease, lymphoma, primary malignancy or metastatic disease, as opposed to large bowel intussusception, which is due to an underlying malignancy in the majority of cases.

Clinical presentation of adult intussusception is typically significant for signs and symptoms of bowel obstruction. The diagnosis is radiologic, with imaging modalities that can be diagnostic or ancillary including plain radiographs, ultrasound, computed tomography scan, and endoscopy. Surgical management of adult intussusception is mandatory, with specific operative intervention tailored to anatomic location and etiology. If the diagnosis of a benign etiology is definitive, distal to proximal reduction can be safely performed followed by limited resection with primary anastamosis [5]. However, when the etiology is unknown or when the presence of malignancy is unequivocal, a formal oncological resection should be performed. This may include a primary anastamosis or stoma formation depending on location, bowel wall integrity, degree of contamination, and surgeon preference.

Although bladder cancers can disseminate hematogenously or lymphatically, superficial tumors (T1) rarely metastasize. When metastatic disease is present, it is most frequently in the pelvic lymph nodes, liver, lungs, or bone [6], with no reported cases of superficial local disease metastasizing to the gastrointestinal tract to date. Even muscle-invading cancers (≥T2), which metastasize more frequently, rarely spread to bowel, accounting for only 13% of all sites of metastatic disease from both transitional cell and squamous cell carcinomas [7].

Transitional cell carcinomas of the genitourinary tract with sarcomatoid differentiation are extremely rare, representing tumors with both epithelial and non-epithelial components. Although debate exists as to the pathogenesis and nomenclature of such tumors, [8] they tend to be more aggressive with a higher incidence of malignancy. Perhaps the natural history of this subtype of transitional cell carcinoma contributed to our patient's unique presentation and pattern of metastasis despite only local invasion of the bladder urothelium.

## Conclusion

A metastatic lesion in the transverse colon, with an atypical invasive pattern, is an unexpected finding following complete resection of a superficial bladder carcinoma. This pathology mandates volume resuscitation and surgical management in a timely fashion. The aggressive nature of sarcomatoid bladder carcinoma presents a challenging clinical situation even after R0 resection has been performed. Reported is the unique case of a superficial sarcomatoid bladder carcinoma with an isolated metastatic lesion to the transverse colon causing intussusception and large bowel obstruction.
